# ALDH1A inhibition sensitizes colon cancer cells to chemotherapy

**DOI:** 10.1186/s12885-018-4572-6

**Published:** 2018-06-15

**Authors:** Z. Kozovska, A. Patsalias, V. Bajzik, E. Durinikova, L. Demkova, S. Jargasova, B. Smolkova, J. Plava, L. Kucerova, M. Matuskova

**Affiliations:** Laboratory of Molecular Oncology, Cancer Research Institute, Biomedical Research Center of SAS, Dubravska cesta 9, 845 05 Bratislava, Slovakia

**Keywords:** ALDH1, Cancer stem cells markers, Chemotherapy, Colon cancer

## Abstract

**Background:**

Recent evidence in cancer research, developed the notion that malignant tumors consist of different subpopulations of cells, one of them, known as cancer stem cells, being attributed many important properties such as enhanced tumorigenicity, proliferation potential and profound multidrug resistance to chemotherapy. Several key stem cells markers were identified in colon cancer. In our study we focused on the aldehyde dehydrogenase type 1 (ALDH1) expression in colon cancer-derived cell lines HT-29/eGFP, HCT-116/eGFP and LS-180/eGFP, and its role in the chemoresistance and tumorigenic potential.

**Methods:**

The effect of pharmacological inhibition of ALDH activity by diethylaminobenzaldehyde (DEAB) and also effect of molecular inhibition by specific siRNA was evaluated in vitro in cultures of human colorectal cell lines. The expression level of different isoenzymes of aldehyde dehydrogenase was determined using qPCR. Changes in cell biology were evaluated by expression analysis, western blot and apoptosis assay. The efficiency of cytotoxic treatment in the presence of different chemotherapeutic drugs was analyzed by fluorimetric assay. Tumorigenicity of cells with specific ALDH1A1 siRNA was tested in xenograft model in vivo.

**Results:**

Treatment by DEAB partially sensitized the tested cell lines to chemotherapeutics. Subsequently the molecular inhibition of specific isoforms of ALDH by ALDH1A1 or ALDH1A3 siRNA led to sensitizing of cell lines HT-29/eGFP, HCT-116/eGFP to capecitabine and 5-FU. On the model of athymic mice we observed the effect of molecular inhibition of ALDH1A1 in HT-29/eGFP cells by siRNA. We observed inhibition of proliferation of subcutaneous xenografts in comparison to control cells.

**Conclusion:**

This research, verifies the significance of the ALDH1A isoforms in multidrug resistance of human colorectal cancer cells and its potential as a cancer stem cell marker. This provides the basis for the development of new approaches regarding the treatment of patients with colorectal adenocarcinoma and potentially the treatment of other tumor malignancies.

## Background

Colorectal cancer (CRC) has been a serious public health problem in developed countries for many years because of its frequency despite the screening and preventive strategies [1]. It remains the third most common cancer in men and the second most common cancer in women [2]. Chemotherapeutic regimens combining: Folinic acid, Fluorouracil and Oxalipatin (FOLFOX), Folinic acid, Fluorouracil and Irinotecan hydrochloride (FOLFIRI), Folinic acid, Fluorouracil, Irinotecan hydrochloride and Oxaliplatin (FOLFIRINOX) or Irinotecan, Fluorouracil and Leucovorin (IFL) still remain key therapeutic modalities in CRC [3–6]. Treatment failure or poor response to the treatment arises either as a result of acquired or intrinsic ability of tumor cells to become simultaneously resistant to a wide range of antineoplastic agents with different structure and mechanism of action. Multidrug resistance is primarily caused by over-expression of ATP-binding cassette transporters (ABCT), efflux pumps that decrease bioavailability of the administered drug(s) [7]. Substantial heterogeneity in tumor mass also contributes to drug resistance. It is widely accepted, that only a small subpopulation of tumor cells drives tumor growth and form metastases. These cells designated as cancer stem cells (CSC) possess capability of self-renewal, differentiation properties and also inherent drug resistance. CSC typically represent 0.1–10% of all tumor cells and they can be identified based on their expression of specific surface markers (CSC makers) [8, 9] such as LGR5 [10–12], CD44v6, [13, 14] CD133 [15] and others.

Aldehyde dehydrogenase (ALDH) is an oxidoreductase, which catalyzes a conversion of aldehydes to their corresponding carboxylic acids [16]. The human genome contains 19 ALDH genes with various cellular functions and tissue distribution [17]. Several ALDH isoforms have been identified as CSC markers in different type of cancer. It was confirmed that there is a correlation between ALDH1 expression and bad prognosis for patients in embryonal rhabdomyosarcoma [18], acute myeloid leukemia (AML) [19], pancreatic adenocarcinoma [20], breast cancer [21], lung cancer [22] and ovarian cancer [23, 24].

Acquired drug resistance in cancer cells is associated with the transcriptional activation of ALDH1 expression. The *ALDH1A1* gene encodes a homotetramer that is ubiquitously distributed in adult organs, such as brain, testis, kidney, eye, lens, retina, liver, and lungs. ALDH1A1 together with ALDH1A2 and ALDH1A3 takes its position among the three highly conserved cytosolic isozymes, which catalyze the oxidation of retinal (retinaldehyde), the retinol metabolite, to retinoic acid (RA) [25]. Despite accumulating evidence on the functional role of ALDH1A1 in normal stem cell and CSC, the specific mechanisms involved in the regulation of ALDH1A1 remain unclear [26]. The ALDH1A1 provides drug protection and radiation resistance to CSCs [26]. This effect was observed on hematopoietic progenitor cells [27].

The present study aims to characterize relationship between expression of ALDH isoforms and resistance to chemotherapeutics used in the treatment of patients with colorectal carcinoma. The role of specific ALDH isoforms in chemoresistance and stemness in colon cancer has not been studied in detail, yet. There is some information about ALDH1B1 isoform which can be a diagnostic marker for colon cancer [28]. For our experiments we explored the role of ALDH1A1 and ALDH1A3 isoforms in human colorectal cell lines HCT-116/eGFP, HT-29/eGFP and LS-180/eGFP. We identified, that ALDH1A1 and ALDH1A3 isoforms are differentially expressed in selected cell lines along with other CSC markers. Silencing the expression by siRNA interference method altered sensitivity to the chemotherapeutics indicating that the specific ALDH isoforms contribute to drug resistance in CRC.

## Methods

### Chemicals

All chemicals were purchased from Sigma Aldrich (St Louis, MO, USA), if not stated otherwise.

### Cell lines

Human colorectal adenocarcinoma cell lines HT-29 (ATCC® Number HTB-38™), HCT-116 (ATCC® Number CCL-247™ and, LS-180 (ATCC® -Number CL-187™) were used in this study. Cells were retrovirally transduced by enhanced Green fluorescent protein gene (eGFP) as described previously in [29] and designed as follows: HT-29/eGFP, HCT-116/eGFP and LS-180/eGFP.

Cells were cultured in high-glucose (4.5 g /L) Dulbecco’s modified Eagle medium (DMEM, PAN Biotech, Germany) supplemented with 5 or 10% fetal bovine serum (FBS, Biochrom AG), 2 mM glutamine or glutamax and antibiotic-antimycotic mix (GIBCO BRL, Gaithesburg, MD).

### Aldefluor assay

To evaluate the ALDH activity, functional ALDEFLUOR™ assay (StemCell Technologies, USA) was performed. The cell suspension was centrifuged for 5 min at 250 x g, the supernatant was removed and the cells were resuspended in 1 ml of ALDFLUOR Assay Buffer. Finally, cell count was performed and the sample was adjusted to a concentration 1 × 10^6^ cells/ml with ALDEFLUOR Assay Buffer. We proceeded according to manufacturer’s protocol.

Before measurement DAPI was added to both control and test tubes to distinguish dead cells.

Measurement was performed using BD FACSCanto™ II flow cytometer (Becton Dickinson, USA) equipped with FacsDiva program. Data were analyzed with FCS Express program.

### RNA isolation and cDNA synthesis

Total RNA was isolated from 1 to 2 × 10^6^ tumor cells by NucleoSpin® RNA II Mini Total RNA Isolation Kit (Macherey Nagel, Germany) according to manufacturer’s protocol. Extra genomic DNA digestion was performed by RapidOut DNA Removal Kit (Thermo Scientific, Germany). RNA was reverse transcribed with RevertAid™ H minus First Strand cDNA Synthesis Kit (Thermo Scientific, Germany). One μg of cDNA was subjected to standard PCR performed in 1× PCR Master Mix (Thermo Scientific, Germany) with 35 cycles on Biorad Thermal cycler T100 (Biorad, USA) and resolved in 2% agarose or 4% MetaPhor® Agarose (Lonza, Rockland, ME, USA).

### Real time PCR

Quantitative PCR was performed in 1× GoTaq® qPCR Master Mix (Promega) 0.16 μM primers and 1 μl of template cDNA on Bio-Rad CFX 96 (Biorad, USA). The PCR run according to the protocol. Obtained data were subsequently analyzed using CFX Manager™ Software (Version 1.5). Gene expression was calculated using delta cycle threshold values (ΔCt = Ct_TARGET GENE_ – Ct_REFERENCE GENE_). Expression of HPRT1 and GAPD genes was set as endogenous reference gene. Analysis was performed twice in triplicates and data were expressed as means ± SD.

Primers used for PCR a qPCR are listed in the Table 1.

**Table 1:** Sequences of primers used for PCR and qPCR

Gene	PCR product size		sequence		
ALDH1A1	182 bp	S	TTGGAATTTCCCGTTGGTTA	62 °C	[19]
		A	CTGTAGGCCCATAACCAGGA		
ALDH1A3	133 bp	S	GCCCTTTATCTCGGCTCTCT	60 °C	[20]
		A	CGGTGAAGGCGATCTTGT		
ALDH2	168 bp	S	ACCTGGTGGATTTGGACATGGTCC	60 °C	[20]
		A	TCAGGAGCGGGAAATTCCACGGA		
LGR5	101 bp	S	ACAGGAAATCATGCCTTACAGAGCTT	60 °C	[21]
		A	ACTCCAAATGCACAGCACTGGT		
CD133	162 bp	S	TTGTGGCAAATCACCAGGTA	60 °C	
		A	TCAGATCTGTGAACGCCTTG		
MDR1	169 bp	S	GCTATAGGTTCCAGGCTTGCT	60 °C	
		A	GTGCTTGTCCAGACAACATTT		
nanog	207 bp	S	GCAAATGTCTTCTGCTGAGATGC	60 °C	
		A	AGCTGGGTGGAAGAGAACACAG		
HPRT1	137 bp	S	TGACCAGTCAACAGGGGACA	62 °C	[22]
		A	ACTGCCTGACCAAGGAAAGC		
GAPDH	226 bp	S	GAAGGTGAAGGTCGGAGTC	58 °C	[23]
		A	GAAGATGGTGATGGGATTTC		

### siRNA nucleofection and gene silencing by siRNA interference

Suspension of 1–2 × 10^6^ tumor cells was transfected with small-interfering RNA (siRNA) oligonucleotide according to the manufacturer’s instructions using the Neon® transfection system (Invitrogen, USA). The parameters used for electroporation are specified in Table 2.

**Table 2:** The parameters used for electroporation

Cell line	Pulse voltage (V)	Pulse width (ms)	Pulse number
HCT-116/eGFP	1300	30	1
HT-29/eGFP	1400	20	2
LS-180/eGFP	1400	20	1

We used 100 μl tips type. Cells were plated immediately after transfection on the 6 wells plate with pre- warmed cultivation medium without antibiotics. After cultivation for 24 H or 48 H cells were harvested and used for sequential analyses.

The siRNA oligonucleotides were purchased from Sigma – Aldrich for ALDH1A1 (EHU028501-50UG, MISSION® esiRNA Human aldh1a1) for ALDH1A3 (MISSION® siRNA SASI_Hs01_00129096 and MISSION® siRNA SASI_Hs01_00129097 mixed 1:1 to reach efficient silencing of ALDH1A3 gene) and negative control (SIC001-10NMOL, MISSION® siRNA Universal Negative Control 1).

### Fluorimetric assay

The analysis was performed in black 96-well plates (Greiner cat number 655090). Depending on the particular cell line 1.10^3^–5.10^3^ cells per well was seeded. Forty eight hours later cells were exposed to various concentrations of either chemotherapeutics alone or their combinations with DEAB. Cytotoxic effect of chemotherapeutic agents on HT-29/eGFP, HCT-116/eGFP and LS-180/eGFP cell lines was measured by fluorimetric assay. Measurements were performed after 5 days of cultivation. Tests were performed in quadruplicates.

### Western blotting

Western blot analysis was performed as described previously [30]. ALDH1–specific antibody (BD Biosciences cat. 611,194) at 1:2000 dilution and anti β-actin antibody (Cell Signaling Technologies, cat. 3700S) at 1:2000 dilution served as a loading control.

### Apoptosis detection

Apoptotic cells were detected by Annexin V staining as described previously [31].

### Xenotransplant growth and animal treatments in vivo

Six to eight weeks old athymic mice (Balb/c nu/nu) were used in accordance to the institutional guidelines under the approved protocols. Tumors were induced by s.c. administration of 2.5 x 10^5^ HT-29/eGFP cells transfected with ALDH1A1 siRNA or negative siRNA cells resuspended in 100 μl of serum-free DMEM. Animals (*n* = 8) were evaluated for tumor growth and size regularly. Tumor volume was calculated from caliper measurements according to formula volume = length width^2^ /2. Results were evaluated as mean tumor volume ± SD.

### Statistical analysis

For the statistical analysis the animal models were divided in two groups: female (*n* = 4) and male (*n* = 4). The normality assumption hypothesis was tested using Shapiro-Wilk test. Differences between two groups in individual time points were assessed by Student’s-t test or Mann-Whitney U test depending on normality of the data. Multivariate analysis was performed using General linear model for repeated measures with Greenhouse-Geisser correction if violation of sphericity was assumed. Effects of molecular inhibition and sex of animal were analyzed. *P* values < 0.05 were considered to indicate statistical significance.

## Results

### Molecular inhibition of ALDH by DEAB sensitizes HT-29/eGFP cells to chemotherapeutic treatment

In order to find the link between the ALDH expression and drug responses in CRC cell lines, first we determined IC_50_ for capecitabine (CAP), raltitrexed (RAL), 5-fluorouracil (5-FU) and irinotecan (IRI). Cell lines exhibited susceptibility to these agents with IC_50_ in similar range (Table 3). DEAB is a non-specific inhibitor of the aldehyde dehydrogenase enzyme activity. It is an inhibitor of MORE than one isozymes of ALDH; and has two pharmacodynamic actions: 1. direct inhibitor for some of the isoforms and 2. slow or very slow substrate for others [32]. We used it in combination with chemotherapeutic treatment (sub-inhibitory concentration of DEAB 20 μg/ml), in order to determine whether the combinatory treatment is more effective. As shown in the Table 4, combination chemotherapy with DEAB resulted in altered chemo-sensitivity when compared to single chemotherapy in HT-29/eGFP cells. In the case of CisPt, DEAB decreased 1.6-fold the IC_50_, in case of RAL it decreased 1.8 fold the IC_50_, in case of 5-FU it decreased 126.7-fold IC_50_ and in case of IRI it decreased 1086-fold the IC_50_.

**Table 3:** IC_50_ values calculated by Calcusyn for 3 CRC cell lines treated by selected chemotherapeutic agents

Chemotherapeutic agent	CRC cell line	IC_50_ value (μg/ml)
CAP [μg/ml]	HCT-116/eGFP	296.13 (*r* = 0.993)
	HT-29/eGFP	309.50 (*r* = 0.921)
	LS-180/eGFP	267.86 (*r* = 0.952)
RAL [μg/ml]	HCT-116/eGFP	2.5 (*r* = 0.866)
	HT-29/eGFP	0.69 (*r* = 0.849)
	LS-180/eGFP	3.48 (*r* = 0.916)
5-FU [μg/ml]	HCT-116/eGFP	0.48 (*r* = 0.959)
	HT-29/eGFP	0.38 (*r* = 0.844)
	LS-180/eGFP	0.27 (*r* = 0.992)
IRI [μg/ml]	HCT-116/eGFP	1.30 (*r* = 0.989)
	HT-29/eGFP	3.26 (*r* = 0.882)
	LS-180/eGFP	0.37 (*r* = 0.943)

The results are from two independent experiments measured in quadruplicates

**Table 4:** IC_50_ values calculated by Calcusyn for single chemotherapy or chemotherapy in combination with DEAB tested on HT-29/eGFP cancer cells

	IC_50_ (μg/ml)
CisPt	0.36 (*r* = 0.985)
CisPt + DEAB (20 μg/ml)	0.22 (*r* = 0.993)
RAL	0.69 (*r* = 0.849)
RAL + DEAB (20 μg/ml)	0.37 (*r* = 0.977)
5-FU	0.38 (*r* = 0.844)
5-FU + DEAB (20 μg/ml)	0.003 (*r* = 0.508)
IRI	3.26 (*r* = 0.882)
IRI + DEAB (20 μg/ml)	0.003 (*r* = 0.750)

The results are from two or three independent experiments measured in quadruplicates

**Fig. 1:**
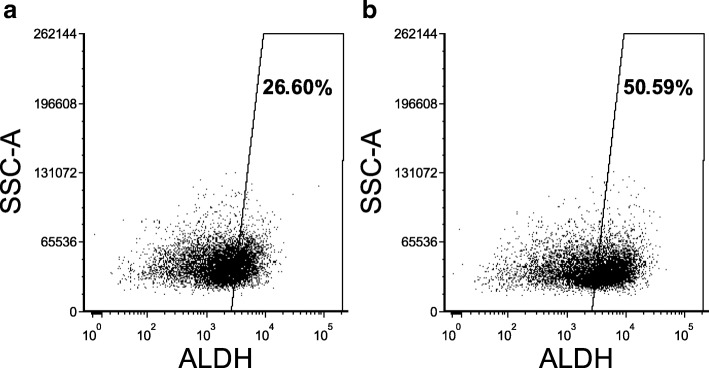
ALDH activity in HT-29/eGFP cells measured by ALDEFLUOR assay by flow cytometry. Cells were treated with 40 μg/ml DEAB 24 h prior measurement (**a**) and compared with untreated samples (**b**)

Subsequently we tested ALDH activity by ALDEFLUOR assay between untreated cells and cells treated with DEAB. These samples were exposed to 40 μg/ml DEAB 24 h prior measurement. As we expected, cells cultivated with DEAB had decreased ALDH activity in comparison with untreated cells Fig. 1. The sample treated with 40 μg/ml DEAB had 1.9-fold lower ALDH activity comparing to untreated sample.

### ALDH isoforms are differentially expressed in tested CRC cell lines

**Fig. 2:**
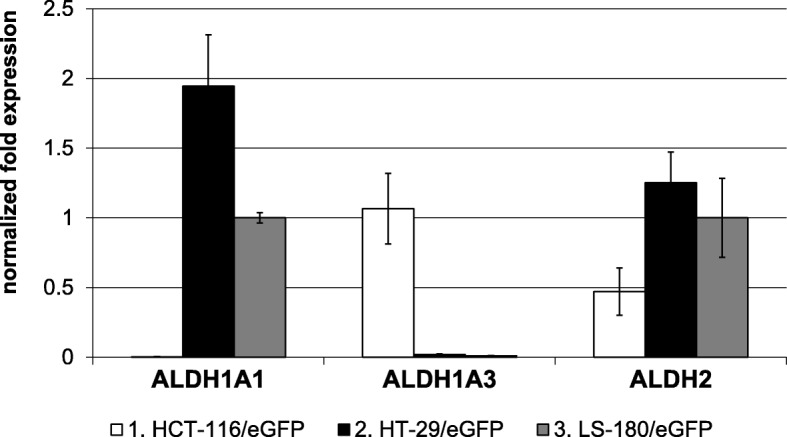
Normalized fold expression of analyzed genes in tested cell lines. RNA was isolated from the tumor cells, reverse transcribed and PCR amplified to detect the gene expression. Data were analyzed using CFX Manager™ Software, Version 1.5. Gene expression was calculated using delta cycle threshold (ΔCt = CtTARGET GENE – CtREFERENCE GENE) values, where HPRT1 and GAPDH expressions were chosen as endogenous reference genes. The cell line LS-180/eGFP was set as control. Analysis was performed in triplicates or quadruplicates in three independent experiments and quantitative data were expressed as means ± SD

**Fig. 3:**
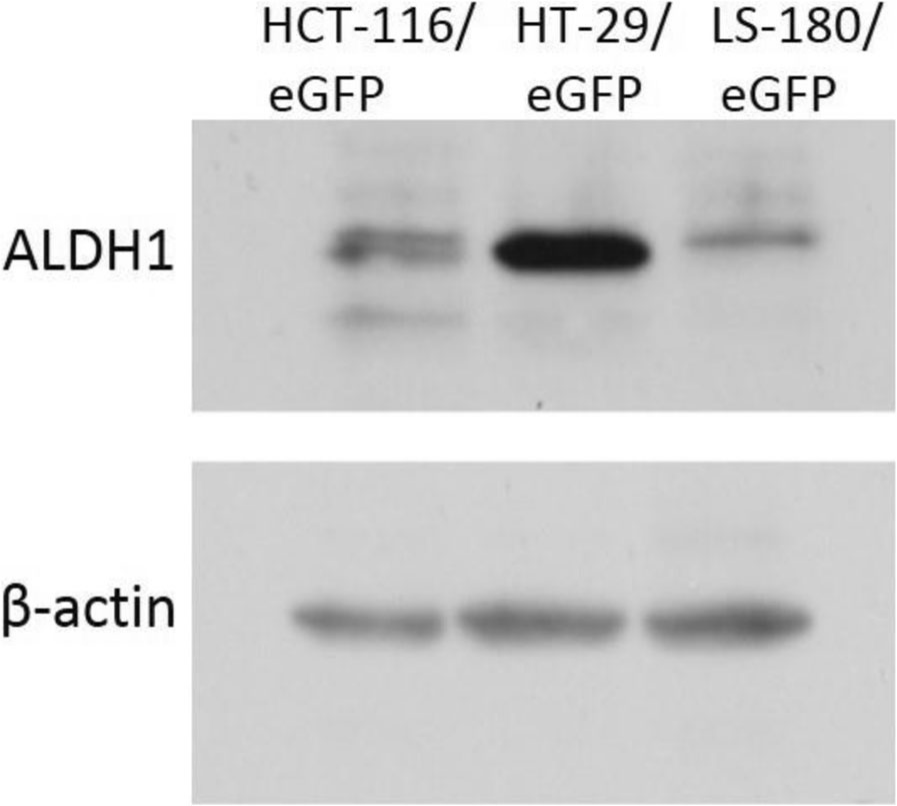
Western blot of protein extracts. Whole protein extracts from HT-29/eGFP, HCT-116/eGFP and LS-180/eGFP were used for Western blot with specific antibodies anti-ALDH1A. Antibody anti-beta Actin was used as loading control

Based on the capability of the ALDH inhibition to sensitize tumor cells to chemotherapy, we examined the expression of specific ALDH isoforms in selected cell lines by qPCR and Western blot. We observed differences in expression of analyzed ALDH isoforms. Cell line HT-29/eGFP dominantly expressed ALDH1A1 isoform, cell line HCT-116/eGFP dominantly expressed ALDH1A3 isoform and cell line LS-180/eGFP expressed in the similar level ALDH1A1 and ALDH2 isoforms (Fig. 2). We detected ALDH1 proteins by Western blot. As it is shown in Fig. 3, this ALDH1 is mainly expressed in HT-29/eGFP cells what is in accordance with qPCR results.

### CSC markers are differentially expressed in tested CRC cell lines

**Fig. 4:**
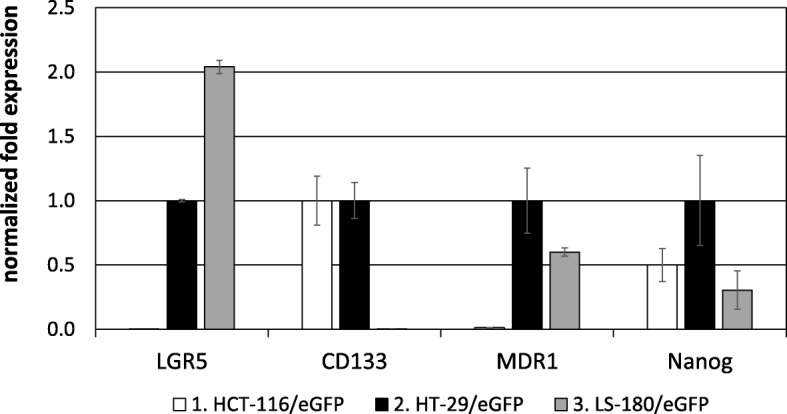
Normalized fold expression of various CSC markers in tested cell lines. RNA was isolated from the tumor cells, reverse transcribed and PCR amplified to detect the gene expression. Data were analyzed using CFX Manager™ Software, Version 1.5. Gene expression was calculated using delta cycle threshold (ΔCt = CtTARGET GENE – CtREFERENCE GENE) values, where HPRT1 and GAPDH expressions were chosen as endogenous reference genes. The cell line HT-29/eGFP was set as control. Analysis was performed in triplicates or quadruplicates in three independent experiments and quantitative data were expressed as means ± SD

We subjected tested cell lines HCT-116/eGFP, HT-29/eGFP and LS-180/eGFP to qPCR for analysis of the expression level of various CSC markers in order to detect possible correlations between the expression of ALDH isozymes and other CSC markers including LGR5, CD133, MDR1 and Nanog. The CSC markers expression verifies the existence of CSC subpopulation in our cell lines and the clinical implications they have. Also the differential expression profile analysis shows that different CSC markers are expressed in each cell line with CD133 and Nanog being expressed mainly in HCT-116/eGFP and HT-29/eGFP cells while LGR5 and MDR1 being expressed in HT-29/eGFP and LS-180/eGFP cells, neither in HCT-166/eGFP (Fig. 4). This fact could account partly, for the difference in response to chemotherapy observed between the two cell lines, since it indicated the differences between the CSC subpopulations for different tumor types. We can correlate the ALDH1 concomitant high expression with the increased resistance in the respective colorectal cancer cell subpopulation - i.e. colorectal cancer stem cells.

### Inhibition of ALDH1 isoforms sensitizes cancer cell lines to chemotherapy

**Fig. 5:**
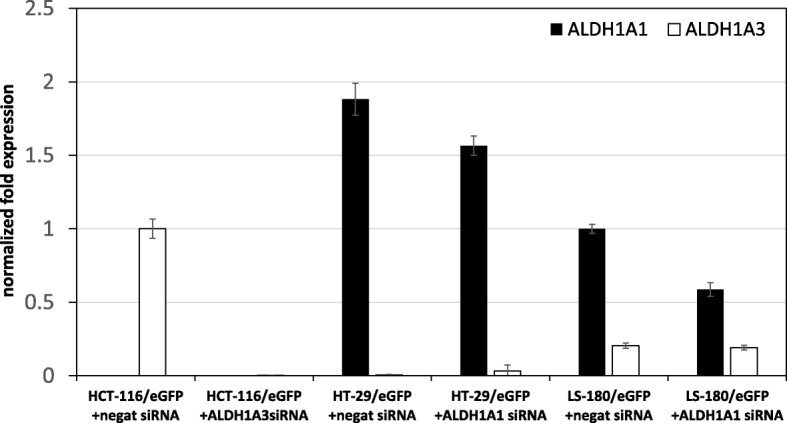
Normalized fold expression of analyzed genes in nucleofected cell lines. RNA was isolated from the tumor cells, reverse transcribed and PCR amplified to detect the gene expression. Data were analyzed using CFX Manager™ Software, Version 1.5. Gene expression was calculated using delta cycle threshold (ΔCt = CtTARGET GENE – CtREFERENCE GENE) values, where HPRT1 and GAPDH expressions were chosen as endogenous reference genes. Analysis was performed in triplicates or quadruplicates in three independent experiments and quantitative data were expressed as means ± SD

Based on the previous results, we performed siRNA silencing of *ALDH1A1* gene in HT-29/eGFP and LS-180/eGFP, and *ALDH1A3* gene in HCT-116/eGFP. We measured efficiency of silencing prior to performing subsequent tests. The efficiency of silencing was confirmed by qPCR and it was different for each cell lines as described: 17% for HT-29/eGFP, 42% for LS-180/eGFP and 99% for HCT-116/eGFP (Fig. 5).

Transfected cells were exposed to previously used chemotherapeutics in order to see the alteration linked to the specific inhibition. Sensitivity of specifically silenced cells was compared to sensitivity of cells which were transfected with negative siRNA only (Table 5). Silenced cells HT-29/eGFP were 3.7-fold more sensitive to CAP and 55-fold more sensitive to 5-FU. Silenced cells LS-180/eGFP were 3.5 fold more resistant to CAP, 4.4 fold more resistant to 5-FU, 13.2-fold more resistant to IRI and they were sensitive only 1.2 fold more to RAL. In HCT-116/eGFP cell line, ALDH1A3 silencing resulted in sensitization effect only in case of 5-FU. These cells were 1.3-fold more sensitive to 5-FU and 3.3-fold more resistant to IRI. The susceptibility of nucleofected cells were measured after 5-day incubation with chemotherapeutic agents, by fluorimetric assay. These results confirm the role of ALDH1 isoform group in the MDR of CRC chemotherapy and further indicated its role as a CSC marker.

**Table 5:** IC_50_ values for used chemotherapeutics calculated by Calcusyn for transfected cell lines

IC50	CAP (μg/ml)	5-FU (μg/ml)	RAL (μg/ml)	IRI (μg/ml)
HCT-116/eGFP				
negat siRNA	296.13 (*r* = 0.993)	0.28 (*r* = 0.985)	1.88 (*r* = 1.000)	0.03 (*r* = 0.971)
ALDH1A3 siRNA	300.00 (*r* = 0.987)	0.21 (*r* = 0.992)	1.79 (*r* = 1.000)	0.10 (*r* = 0.924)
HT-29/eGFP				
negat siRNA	240.34 (*r* = 1.00)	0.044 (*r* = 0.997)	0.83 (*r* = 0.998)	1.43 (*r* = 0.999)
ALDH1A1 siRNA	64.27 (*r* = 0.955)	0.0008 (*r* = 0.866)	0.81 (*r* = 0.998)	1.53 (*r* = 0.998)
LS-180/eGFP				
negat siRNA	267.86 (*r* = 0.952)	0.70 (*r* = 0.454)	2.06 (*r* = 1.000)	0.262 (*r* = 0.893)
ALDH1A1 siRNA	927.74 (*r* = 0.999)	3.07 (*r* = 0.986)	1.76 (*r* = 1.000)	3.46 (*r* = 0.998)

The results are from one experiment measured in quadruplicates

**Fig. 6:**
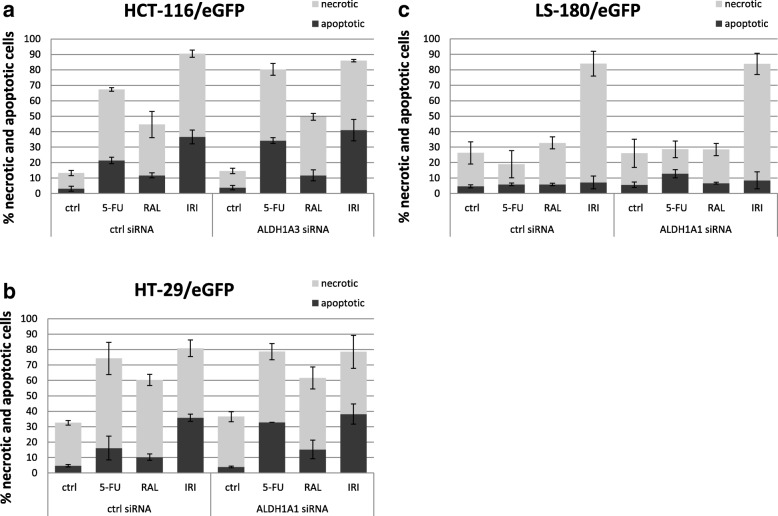
Induced apoptosis and necrosis in cells transfected with specific siRNA measured by Annexin V assay. Concentrations of chemotherapeutics used in this assay were as follow: **5-FU**: 5 μg/ml for HCT-116/eGFP, 20 μg/ml for HT-29/eGFP and LS-180/eGFP; **RAL**: 20 nM for HCT-116/eGFP and HT-29/eGFP, 50 nM for LS-180/eGFP; **IRI**: 20 μg/ml for HCT-116/eGFP and HT-29/eGFP, 25 μg/ml for LS-180/eGFP. **a**: HCT-116/eGFP, **b**: HT-29/eGFP, **c**: LS-180/eGFP

In order to confirm the data from the viability assays, we performed also Annexin V assay for detection of apoptosis and necrosis in the cells transfected with specific siRNA influenced by different chemotherapeutics (Fig. 6). Silencing of ALDH1A3 in HCT-116/eGFP increased the proportion of apoptotic cells upon 5-FU treatment from 21.46 to 34.21%, upon IRI treatment from 36.61 to 41.02%. The proportion of necrotic cells upon RAL treatment was increased from 32.9 to 37.82% and decreased upon IRI treatment from 53.92 to 44.97%. Silencing of ALDH1A1 in HT-29/eGFP increased the proportion of apoptotic cells upon 5-FU treatment from 16.11 to 32.9%, upon RAL treatment from 10.26 to 15.22% and upon IRI treatment from 35.76 to 38.19%. Silencing of ALDH1A1 in LS-180/eGFP increased the proportion of apoptotic cells upon 5-FU treatment from 5.89 to 12.82% upon RAL treatment from 5.86 to 6.70% and upon IRI treatment form 7.12 to 8.51%. The portion of necrotic cells increased upon 5-FU treatment from 13.04 to 15.78% and upon RAL treatment the portion of necrotic cells decreased from 26.86 to 21.73%. In case of other chemotherapeutic there were only slight differences in percentage of apoptotic and necrotic cells. We conclude that inhibition of specific ALDH isoform in the cancer cells substantially affected their responses to multiple chemotherapeutic drugs.

### ALDH1A1 silencing decreases tumorigenicity of the HT-29/eGFP cells on athymic mice

**Fig. 7:**
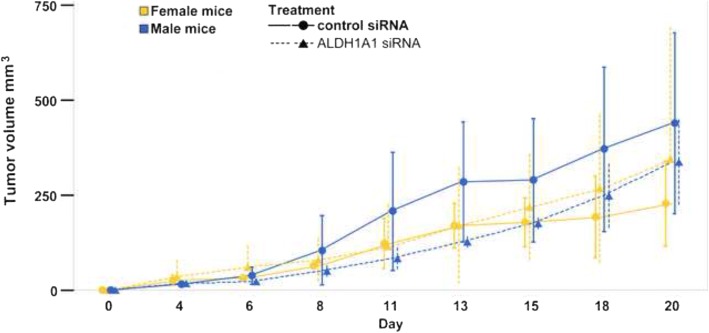
The tumor volume recordings from Balb/c nu/nu mice models for in vivo experiments. Tumors were induced by s.c. administration of 2.5 10^5^ HT-29/eGFP cells transfected with ALDH1A1 siRNA or negative siRNA cells resuspended in 100 μl of serum-free DMEM. Animals (*n* = 8) were divided into two groups females (*n* = 4) and males (*n* = 4). The tumor volume was measured, duly and carefully, from 4th day post inoculation. The mice were screened for tumor growth and the tumor volume was calculated according to the formula: volume = length x width^2^/2

In order to examine the role of ALDH1A1 in tumorigenesis, ALDH1A1 silenced HT-29/eGFP cells and negative siRNA transfected HT-29/eGFP cells were injected subcutaneously. Tumor volume was measured regularly for subsequent 20 days. Animals were sacrificed, when the tumors exceeded 1 cm in one dimension. The results unraveled a decrease in the tumor growth in the xenografts derived from ALDH1A1-silenced cells in comparison to the respective xenografts from cells nucleofected with negative siRNA. From day 8 of the study the measurement shows a decrease of 23.86%, on day 11 the decrease in growth rate is 40.5%; on day 13 it is 34.8% and on day 15 the difference in growth rate is 15.83%. Finally, on day 20 the difference in the tumor volume began to decrease with a percentage difference of 8.7%. For the statistical analysis the animals were divided in two groups: female (*n* = 4) and male (*n* = 4). Multivariate analysis of repeated measures showed no differences in tumor size between tumors induced by cell nucleofected with negative siRNA and ALDH1A1 siRNA, even when stratified by sex (*P* = 0.119). However, for male mice only, *P* value reach borderline significance *P* = 0.097 with significant difference in tumor size for days 11 and 13 (*P* = 0.047 and 0.019, respectively) Fig. 7.

In males RA production is dependent on both ALDH1A1 and ALDH1A3, however in females ALDH1A3 is the predominant enzyme for RA production [33, 34]. The sex hormones regulate tissue-specific pattern of ALDH1 enzyme expression [35].

## Discussion

Chemotherapy remains one of the major pillars in treatment of colorectal carcinoma. The drug regimens used for the disease control comprise 5-fluorouracil, oxaliplatin, capecitabine, irinotecan and raltitrexed. Even though these agents exert their cytotoxic effect by multiple non-overlapping mechanisms, tumor often develops resistance to these drugs which represents major clinical problem. Better understanding of the drug resistance mechanisms and development of the agents, which could target these mechanisms, is still needed. It has been postulated that there is a tight link between the phenotype of cancer stem cells and chemoresistance. The cytotoxic treatment targets mostly rapidly dividing tumor cell subpopulations thereby leaving behind predominantly the quiescent dormant cells with high chemoresistance often correlating with enrichment for the cancer stem cell markers. Several surface markers were associated with the CSC population in colorectal cancer, such as CD133, CD44v6, Lgr5 [15]. However, CSC surface markers identified so far are expressed also by normal SCs, preventing their potential use as therapeutic targets. In contrast to these surface marker molecules, the ALDH marker represents an intracellular protein with an enzymatic function executing the oxidation of both endogenously and exogenously produced aldehydes to their respective carboxylic acids. High activity of aldehyde dehydrogenase interfere with several chemotherapeutics used in the treatment of patients with colorectal cancer. Detoxification and drug inactivation represents one of the mechanisms contributing to chemoresistance. Overexpression of ALDH1A1 and ALDH3A1 isoforms have been shown to result in greater inactivation of cyclophosphamide in breast cancer [36]. Proportion of ALDH1-positive breast cancer cells was significantly higher in patients after paclitaxel and epirubicin-based chemotherapy [21]. It has been also postulated that the tumor cell population with high ALDH activity is enriched for the CSC, as the enzymatic detoxification renders these cells more resistant to various agents. As the ALDH enzymatic activity may increase survival of CSC in colorectal carcinoma, it might be beneficial to inhibit its activity in order to target the CSC population that remains unaffected by standard chemotherapy.

Based on the wide biologic action of ALDH enzyme, there were efforts to develop inhibitors for the clinical use. Even though there are 14 different inhibitors of the ALDH enzyme superfamily available so far with varying specificities for the particular isoforms, pharmacological inhibitors have been developed for only 3 of the 19 ALDH isozymes. These are the drugs used for the inhibition of enzymes involved in the metabolism of alcohol (ALDH2) and the anticancer oxazaphosphorine drugs specific for the ALDH1A1 and ALDH3A1 isoforms [37].

In our study we investigated the potential role of ALDH in chemosensitivity and tumorigenicity of these cells lines. There was a substantial subpopulation exhibiting ALDH activity as determined by functional Aldefluor assay (Fig. 1), therefore we investigated its contribution to drug responses in detail in three colorectal carcinoma cell lines (HCT-116/eGFP, HT-29/eGFP, LS-180/eGFP). Exposure to the chemotherapeutics used in treatment of colorectal cancer such as 5-FU, CAP, RAL and IRI unraveled differences in sensitivity to these drugs attributable to many potential mechanisms of inherent drug resistance [7]. We identified specific ALDH isoform expression (ALDH1A1 and ALDH1A3) in tested cells. ALDH1A3 isoform was most prominently expressed in HCT-116/eGFP cell line. HT-29/eGFP and LS-180/eGFP cells expressed ALDH1A1 isoform. Since there were different ALDH1 isoforms expressed in the tested cell lines, we decided to further evaluate their role in chemoresistance using siRNA-mediated gene silencing. We could not employ pharmacological inhibition due to the fact, that there is not specific inhibitor of the isoform ALDH1A3 available yet. A similar experiment was performed on melanoma cells, where ALDH1A silencing increased chemosensitivity of these cells [38]. By silencing of ALDH1A1 or ALDH1A3 in tested cells we were compared chemosensitivity of these cells to unaffected ones. Samples after silencing were exposed to 5-FU, CAP, RAL and IRI. Silencing of these two ALDH1A isoforms led to different effect on chemosensitivity to tested drugs. Despite the low efficiency of silencing in cells HT-29/eGFP, the transfection sensitized cells to CAP and 5-FU. Cell line LS-180/eGFP transfected with ALDH1A1 siRNA was more resistant than cells transfected only with negative siRNA. In contrast to this, ALDH1A3 siRNA sensitized HCT-116/eGFP cell lines to raltitrexed and 5-FU. Recent experiments indicate that isoform ALDH1A3 significantly contributes to Aldefluor-positivity in different types of cancer [39, 40]. ALDH1A3 siRNA silencing slightly increased chemosensitivity of HCT-116/eGFP cells. These results confirm that ALDH1A3 may contribute to chemoresistance of colorectal carcinoma cells.

Our data indicate cell line-specific function and role of ALDH isoforms, however it seems reasonable to look for strategies how to specifically target these enzymes in order to improve the cytotoxicity of standard clinical regimens. More importantly, here we show the link between ALDH1A1 and tumorigenic potential of cancer cells. We were able to demonstrate a decrease in tumorigenicity upon ALDH1A1 silencing. Even though the Aldefluor functional assay has shown, that less than 10% of the cells are Aldefluor-positive thus exhibiting ALDH activity, it is sufficient to inhibit this enzyme to alter tumorigenicity indicating its contribution to population of tumor-initiating cells.

We demonstrated that overall ALDH activity in HT-29/eGFP cells decreased after exposure to non-specific inhibitor DEAB. Chemosensitivity test in the presence of subinhibitory concentration of DEAB (20 μg/ml) confirmed its potential to increase the antiproliferative effect of 5-FU, IRI, RAL and CisPt in colorectal carcinoma cells. The effect of specific siRNA (ALDH1A1 or ALDH1A3) is specific only to this isoform. The effect of DEAB is more complex, it effects not only the activity of ALDH but also it has its own cytotoxic effect. These data further support the need for specific ALDH inhibitors to achieve higher cytotoxic action of standard drugs in order to achieve better control of the tumor growth patients. However, further knowledge needs to be gained to thoroughly assess efficacy and safety of this type of treatment.

## Conclusion

Our results confirm the significance of ALDH1 as CSC marker in colorectal carcinoma. We sensitized the cells by pharmacological inhibition with DEAB and also by gene silencing with siRNA. The silencing resulted also in lower tumorigenicity in vivo. However, this effect depends on cell line and each cell line has different expression of ALDH1 isoforms. Taken together our data support the rationale for the development of specific ALDH inhibitors for anticancer therapy.

## Abbreviations

5-FU: 5-fluorouracil
ABCT: ATP-binding cassette transporters
ALDH: Aldehyde dehydrogenase
CAP: Capecitabine
CisPt: Cisplatin
CRC: Colorectal cancer
CSC: Cancer stem cells
DEAB: Diethylaminobenzaldehyde
IRI: Irinotecan
N: Diethylaminobenzaldehyde
RA: Retinoic acid
RAL: Raltitrexed
siRNA: Small-interfering RNA
